# Determinants of health-promoting behavior among eHealth consumers in South Korea: a longitudinal path analysis

**DOI:** 10.7586/jkbns.24.017

**Published:** 2024-08-22

**Authors:** Hanna Choi, Meiling Jin

**Affiliations:** 1 Department of Nursing Science, Nambu University, Gwangju, Korea; 2 Korea University Ansan Hospital, Ansan, Korea

**Keywords:** Health promotion, Consumer health information, Health behavior

## Abstract

**Purpose:**

The study aimed to determine the key factors influencing health-promoting behavior and the behavioral intentions of eHealth consumers based on the health promotion model and technology acceptance model.

**Methods:**

This research involved a longitudinal path analysis. The study was conducted with 360 eHealth consumers aged over 18 years, employed in the top five categories of the Korean standard classification of occupations, and living in the five largest cities in South Korea. The data were analyzed using SPSS 22.0 and AMOS 25.0.

**Results:**

Health-promoting behaviors were directly supported by prior health-related behavior and behavioral intention, and indirectly supported by perceived ease of use, perceived usefulness, perceived benefit, self-efficacy, and behavioral intention. These variables accounted for 36.3% of the variance in health-promoting behavior.

**Conclusion:**

The findings serve as a framework that can help health professionals and health information providers understand how to encourage consumers using eHealth to engage in health-promoting behaviors.

## INTRODUCTION

The coronavirus disease 2019 (COVID-19) crisis has brought enormous changes to numerous citizens’ daily lives [1]. In particular, consumers try to acquire health related information online when they have a health related problem [2]. Obtaining information online regarding health care-related tasks such as searching for the signs and symptoms of a disease, learning about someone else’s experience, finding the name of a hospital, or discovering aftercare information before visiting the doctor is easy. Thus, online health information (OHI) benefits consumers’ self-care and their valuable decision-making process [1].

In South Korea, 91.5% of the population uses internet services, ranking first among 157 nations in the global Information and communications technology development index. This underscores the country's significant reliance on information technology [3]. Thus, the data on Korean consumers reflects the phenomenon of increasingly seeking OHI and using eHealth to participate more in health care management.

The World Health Organization describes eHealth as the efficient and secure application of information and communication technologies to support health and health related activities. This includes the provision of healthcare services, health monitoring, medical literature, health education, as well as the dissemination of knowledge and research [4].

The advancement of information and communication technology (ICT) positively impacts health promoting behaviors (HPBs) by increasing access to health information, encouraging behavioral changes, enabling personalized health management, providing remote healthcare services, and offering psychological support. These changes enhance individuals' health management capabilities and contribute to the improvement of overall health status [5].

There has been increased research on consumers’ use of OHI to investigate this recent trend [2,6,7]. Unfortunately, despite the great debate on OHI’s effects, it is challenging to find quantified indicators about health and the effect OHI has on the health promotion of consumers. The effect of OHI on actual HPB needs to be investigated [8]. Previous research has mainly pertained to understanding individuals’ healthcare–seeking behavior, such as using web portals or web tools [9,10]. These have focused mainly on behavioral intention, present behavior, or short-term changes [11,12]. Predictive research on behavioral models considers various factors, but often excludes the information factor when evaluating the influence of health information [13]. Regarding behaviors associated with health promotion, prior studies have focused on intention [14], and lifestyle changes [15]. Little is known about HPB related to OHI seekers and eHealth consumers. Thus, a systematic review has also pointed out that additional research is required to determine the influences of real health related behavior [16].

The connections established between health related behaviors, technology usage, and health promotion outcomes provide a solid basis for integrating these findings into the theoretical framework of basic nursing science. This integration enhances the understanding of how eHealth tools can be effectively utilized to foster better health outcomes, aligning with core principles of fundamental nursing practice and education. This study can significantly contribute to the development of biological and physiological nursing knowledge by providing insights into how eHealth consumers engage with health information over time and the subsequent impact on their health-promoting behaviors.

### Theoretical background

To predict the usefulness and accuracy of health information obtained online for consumers' HPB, this study is grounded in the health-promotion model (HPM) [17] and the technology acceptance model (TAM) [18].

The TAM is well known in technology-related research. However, despite the increased recognition of the importance of OHI during the COVID-19 pandemic, its significance in terms of health promotion or health behavior has not been fully addressed. The HPM provides useful ideas in terms of prevention and promotion, and studies have included the perceived benefits and self-efficacy variables. However, the HPM has received little validation among eHealth consumers. Using both models, the study predicts the health-promotion behavior of eHealth consumers.

Prior health related behaviors included in HPM are considered important to form habits so that previous behaviors are coded and memorized and so that they can naturally participate in future behaviors without much effort [19]. Prior health related behaviors naturally induce behaviors with healthy lifestyle habits that have been maintained so far, and they directly affect perceived benefits and self-efficacy. In addition, they affect intentions and HPBs directly or indirectly [17,20].

As a result of identifying HPBs and related factors, we have found that prior health related behaviors of Iranian women [19] and female worker [21] were related to perceived benefits, self-efficacy, behavioral intentions, and HPBs. Yang and Kim [22] and Lee [23] confirmed the relationship between prior health related behaviors and perceived benefit as a motivator, and Lee [23] and Shin et al. [24] confirmed perceived efficacy as a motivator.

Perceived ease of use and perceived usefulness, pivotal components in the TAM, have been extensively utilized as factors to elucidate behavioral intention and actual behavior in relation to technology utilization [25,26]. Perceived ease of use is defined as the degree to which a consumer believes that using OHI would be free from effort, and perceived usefulness is defined as the degree to which a consumer believes that using OHI would enhance his or her performance. The TAM is widely accepted because, in contemporary society, consumers tend to depend on technology to seek useful health information themselves rather than depending on passive methods (TV, newspaper etc.) [18,27].

On the other hand, perceived ease of use and perceived usefulness as an antecedent not only lead to perceived benefit, which facilitates health behavior, but also affect HPBs through the medium of self-efficacy [17,28]. Similarly, behavioral intention has been proven to have effects on perceived benefit [24,29] and self-efficacy [30,31].

Perceived benefit has been commonly employed to denote individual motivations. In this study, we also focused on personal motivation, which is more likely to change within the individual dimension. Consumers anticipated benefits from engaging in health behaviors. This is because consumers tend to maintain health behavior longer when they realize that health information benefits them [32]. Perceived self-efficacy of HPB is a mediating factor. Because HPB is the final goal, we focused on the self-efficacy of health behavior in the study rather than internet or technology efficacy. This is a construct of the HPM [17] and has been proven to be the most important predictor of lifestyle changes in meta-analyses [33,34].

Finally, with regard to behavior, behavioral intention refers to a specific plan of action, including when, where, and how, for an organized health behavior [17]. In addition to the HPM, several theories including the Theory of Planned Behavior, the Theory of Reasoned Action, and the TAM have demonstrated that behavioral intention is a significant predictor of health behavior [25,35].

**Figure 1.**
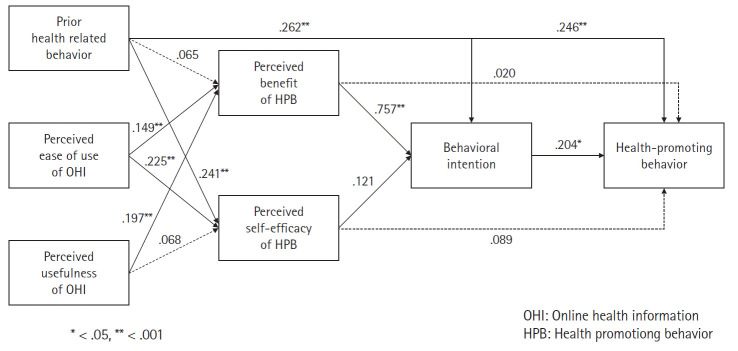
Path diagram of HPB for consumers of OHI .

Based on the studies mentioned above, Figure 1 outlines the identified predictors. Prior health related behavior has an effect on perceived benefit, perceived self-efficacy, behavioral intention, and HPB. Perceived ease of use and usefulness impact perceived benefit and perceived self-efficacy, which in turn affect behavioral intentions and HPB. Additionally, there is a direct relationship between behavioral intentions and HPB.

The objective of this study is to investigate the effect of information utilization on the intentions and behaviors associated with health promotion among eHealth consumers who use OHI , within the frameworks of HPM and TAM.

## METHODS

### 1. Study design

This study is a longitudinal path analysis and tested the factors affecting the behavioral intention and HPB of eHealth consumers based on the HPM and the TAM.

### 2. Participants

The subjects of this study were OHI consumers aged 18 years and older who had sought OHI at least once a month. Participants (n = 360) were recruited using the convenience sampling method. The inclusion criteria were selected using the following sampling: 1) male or female adults who understood the purpose of this study and agreed to participate voluntarily in it; 2) people who had at any time sought OHI (through a webpage or application on a mobile phone or PC) regarding health promotion on topics such as health responsibility, physical activity, nutrition, spiritual growth, self-actualization, stress management, and interpersonal influences within the previous month.

### 3. Instruments

#### Prior health related behavior

Prior health related behavior assesses whether consumers engaged in HPBs during the 6 months preceding the initial survey [17]. The measurement tool for prior health related behavior was adapted from the modified scale for prior health related behavior [37,38]. The instrument encompassed dimensions such as health responsibility, fitness, nutrition, spiritual growth/self-actualization, stress management, and interpersonal influences. Participants rated six items based on their activities over the past 6 months using a 5-point Likert scale. A higher score indicated a higher level of prior health related behavior. In terms of reliability, Cronbach's alpha was reported as .74 in the study conducted by Yun and Park [38], while in this study, the computed Cronbach's alpha was .63, which is the acceptable value [39].

#### Perceived ease of use of OHI

The perceived ease of use of OHI is assessed in terms of its convenience for health management [18]. The perceived ease of use of OHI was utilized by referencing Choi and Jeong [38] and Yun and Park’s [37] modified scale for perceived ease of use, which was originally developed by Davis [18] in the TAM. The instrument consisted of four items rated on a 5-point Likert scale. A higher score indicated a stronger belief among consumers that they would easily accept an information system when seeking health information. In terms of reliability, Cronbach’s alpha was reported as .89 in Yun and Park’s [37] study, while it was computed as .88 in this study.

#### Perceived usefulness of OHI

Perceived usefulness of OHI refers to the degree to which consumers can effectively utilize it for health management. The measurement of perceived usefulness of OHI was adopted with reference to Choi and Jeong [38] and Yun and Park’s [37] modified scale for perceived usefulness, which was derived from Davis [18]. Initially consisting of five items on a 5-point Likert scale, one item related to seeking health information from books and television programs was excluded due to its irrelevance, resulting in four items for analysis. A higher score indicated a stronger belief among consumers that using health information enhances their behavior and decision-making outcomes related to health promotion. In terms of reliability, Cronbach’s alpha was reported as .89 in Yun and Park’s [37] study, while it was computed as .79 in this study.

#### Perceived benefit of HPB

Perceived benefit of HPB measures the advantages that consumers perceive when using OHI [17]. This measurement of perceived benefit of HPB was employed with reference to the modified scale of HPB by Seo and Hah [40]. The instrument originally consisted of six items on a 5-point Likert scale; however, one item with a factor loading below 0.8 was excluded, resulting in five items for analysis. A higher score indicated a stronger motivation among consumers to engage in positive health behavior and a greater likelihood of carrying out HPB for over 6 months. In terms of reliability, Cronbach’s alpha was reported as .90 in Seo and Hah [40], while it was computed as .90 in this study.

#### Perceived self-efficacy of HPB

Perceived self-efficacy of HPB measures individuals' confidence in their ability to manage their health using the OHI they seek [17]. The measuring instrument for perceived self-efficacy of HPB was a revised Korean adaptation of the general self-efficacy scale developed by Lee et al [41]. The instrument comprised three items, each rated on a 5-point Likert scale. When consumers experienced more positive emotions and demonstrated better judgment regarding their ability to complete a task, their sense of self-efficacy became stronger; increased self-efficacy corresponded to lower perceived disturbance. The original scale had a Cronbach's alpha of .75, while in this study, Cronbach's alpha was computed as .71.

#### Behavioral intention

Behavioral intention assesses health responsibility, physical activity, nutrition, spiritual growth, stress management, and interpersonal relations [17]. In this study, behavioral intention corresponds to a commitment to a plan of action within the HPM, signifying a consumer's intent to engage in HPB at a specific time and place, regardless of circumstances. The measuring instrument employed for behavioral intention considered the characteristics of OHI consumers, using a modified scale by Seo and Hah [40]. This instrument consisted of six items rated on a 5-point Likert scale. A higher score denoted a more robust intention to engage in HPB, encompassing improvements in health responsibility, physical activity, nutrition, spiritual growth and self-actualization, stress management, and interpersonal influence. The original scale had a Cronbach's alpha of .80, while in this study, Cronbach's alpha was computed as .78.

#### HPB

HPB gauges the extent of engagement in health responsibility, physical activity, nutrition, spiritual growth, stress management, and interpersonal relations [17]. In relation to HPB, we utilized an adapted version of the Health Promotion Lifestyle Profile-II by Seo and Hah [40], originally developed by Pender et al. [17]. within the HPM. The initial instrument encompassed 50 items, comprising eight items related to health responsibility, eight to physical activity, nine to nutrition, nine to spiritual growth and self-actualization, eight to stress management, and eight to interpersonal influences. Following meticulous review by two nursing professors and two public health professors, the instrument was revised and condensed to 20 items. This revised version included three items on health responsibility, four on physical activity, four on nutrition, three on spiritual growth and self-actualization, three on stress management, and three on interpersonal influences. Confirmatory factor analysis on this modified version yielded no factor loadings below 0.80. A 5-point Likert scale was employed, with a total score ranging from 20 to 100, higher scores indicating greater engagement in HPB. The instrument's Cronbach's alpha was .92 in Seo and Hah [40], while in this study, Cronbach's alpha was computed as .78.

### 4. Data collection

Upon receiving approval from the institutional review board at the researcher's institution, the collected data guided the research which adhered to the principles outlined in the Declaration of Helsinki and followed ethical guidelines. Before obtaining participants' consent and administering the online questionnaires, the researchers provided an explanation of the study's objectives. The sample size was set at 360 individuals, as it is desirable to extract a sample of around 200 to 400 individuals for Structural Equation Modeling using Maximum Likelihood Estimation [36]. Longitudinal data were collected from the five main cities in South Korea—Seoul, Daejeon, Daegu, Busan, and Gwang-ju. The baseline data collection took place from June 10 to 20, 2015, and follow-up data were collected from September 10 to 22, 2015. Three months after the initial survey, respondents were surveyed again to assess various aspects including their online mobile information searches, primary health information services and devices used, inconveniences experienced, and the impact on their HPBs.

### 5. Statistical analysis

General characteristics of the participants and each variable were analyzed using descriptive statistics. The descriptive statistics with normal distribution were expressed by mean ± standard deviation (M ± SD). Skewness and kurtosis were analyzed to ensure normal distribution of variables. Instrument reliability was tested using Cronbach’s alpha. To assess construct validity, convergent validity was tested by exploratory factor analysis and confirmatory factor analysis using principal component analysis. Multicollinearity among variables was analyzed using Pearson’s product moment correlation coefficient. Twelve insincere responses were removed from the 372 collected three months after the initial survey, resulting in a total of 360 responses used for analysis. Missing data were handled by expectation–maximization in SPSS 22.0 (IBM Corporation, Armonk, NY, USA). Model verification was done using maximum likelihood with AMOS 25.0 (IBM Corporation, Armonk, NY, USA). To evaluate model fit, we applied chi-square; normed chi-square; absolute fit indices, which included GFI (goodness-of-fit index), AGFI (adjusted GFI), and RMSEA (root mean square error of approximation); increment fit indices, which included NFI (normed fit index) and CFI (comparative fit index); and parsimony of fit indices, which included PNFI (parsimony NFI). It was considered statistically significant when a two-tailed probability value was less than 0.05.

### 6. Ethical considerations

This study was approved by the Institutional Review Board of the College of Nursing at Seoul National University in Korea (IRB No. E1603/002-004). Detailed informed consent procedures were followed to ensure that all participants fully comprehended the study's purpose, benefits, research questions, and participation process. Participation in the study posed no harm or risk to the participants. Additionally, they were assured that all collected data would be treated confidentially, and they retained the right to withdraw their data at any point. The data were anonymized to prevent the identification of individuals from the survey data, thereby ensuring both the anonymity and confidentiality of the participants. Furthermore, participants were informed that their personal information would be immediately discarded after the study's conclusion, while data devoid of personal identifiers would be securely stored on a locked computer in the laboratory for a period of 5 years.

## RESULTS

### 1. Characteristics of participants

The general characteristics of the 360 respondents were analyzed, including age, sex, occupation, level of education, household income/month in KRW 10,000, cohabitation, and number of chronic diseases (Table 1).

### 2. Use of health information technology in health promotion

Consumers mainly use a PC (44.7%, n = 161) or mobile device (55.3%, n = 199) one or two times a week (27.8%, n = 100) with two or three apps (50.3%, n = 181). The categories of health information most sought were related to health responsibility (39.7%, n = 143) and physical activity (32.8%, n = 118). The purposes of using services were seeking health information (69.4%, n = 250) and searching for materials (10.0%, n = 36). Inconvenient factors in using health information were low credibility (50.4 %, n = 181) and too much information (24.8%, n = 89; Table 1).

### 3. Endogenous variables

As endogenous variables, the average score for prior health related behavior was 18.07 (SD = 3.72), that for perceived ease of use was 11.68 (SD = 2.02), and that for perceived usefulness was 13.78 (SD = 2.81), within a score range from 4 to 20 for each item. The average score for perceived benefit was 15.96 (SD = 5.96) within a score range from 5 to 25, that for self-efficacy was 10.91 (SD = 1.77) with a range from 3 to 15, that for behavioral intention was 21.61 (SD = 3.47 ) with a range from 6 to 30, and that for HPB was 65.07 (SD = 11.18 ) with a range from 20 to 100 (Table 2).

### 4. Path analysis

As a result of testing univariate normality, the absolute value of skewness of all variables was from -.64 to .06, less than 2, and the absolute value of kurtosis was from -.09 to .34, less than 7, satisfying the assumption of univariate normality. Therefore, the data set was adequately modeled by a normal distribution because univariate normality is a necessary condition for multivariate normality. The parameters that fit the information were estimated utilizing the maximum likelihood method. Before model verification, correlations among variables and multicollinearity were examined. The absolute values of the correlation coefficients in this study were from .09 to .51, whereas a correlation of above .7 is generally considered to be a very strong correlation. Thus, there was no multicollinearity problem.

As a result of testing the significance of hypothetical paths and the goodness-of-fit of the hypothetical path model, both were found to be satisfactory. Nine out of 13 OHI consumers’ HPB paths were found to be significant. The fit indices were computed as follows: χ^2^ = 7.92, GFI = .99, AGFI = .94, Normed χ^2^ =2.64, RMSEA = .068, NFI = .99, CFI = .99. The fit indices of the hypothesis model satisfied all fit criteria except PNFI.

Nine out of 13 paths of the model were significant. The squared multiple correlations (SMCs), which indicate the explanatory power of behavioral intention and HPB as consequence variables, were 10.4% and 15.3%, respectively. The SMC of perceived self-efficacy, a factor affecting the consequence variables, was 15.2% (Table 3).

Perceived ease of use and usefulness directly and positively inﬂuenced perceived benefit, and prior health related behavior and Perceived ease of use directly and positively inﬂuenced perceived self-efficacy. Prior health related behavior, perceived benefit, and self-efficacy directly inﬂuenced behavioral intention. Prior health related behavior and behavioral intention directly inﬂuenced HPB, whereas perceived ease of use, perceived usefulness, perceived benefit, and perceived self-efficacy indirectly inﬂuenced HPB (Table 3).

## DISCUSSION

The research serves two primary objectives. Firstly, this study validated factors affecting HPB based on the HPM [17] and the TAM [18]. Through this investigation, we identified that prior health related behavior, perceived benefit, perceived ease of use, perceived usefulness, perceived self-efficacy, and behavioral intention significantly affect HPB. Secondly, by revealing specific predictors for e-health consumers' HPB, we empirically substantiated the behavioral changes among adult consumers further than behavioral intention.

Among exogenous variables, prior health related behaviors were uniquely identified as exerting significant indirect or direct influence on both the behavior intention and HPBs. This finding aligns with conclusions drawn from prior research [20,42]. This study verified the alternative model of patient health engagement in the context of e-health consumers [43], the premise that patients act in response to their thoughts and emotions. Psychosocially, individuals are more influenced by others’ previous actions than their cognitive and emotional states.

In other words, emphasizing prior health related behaviors can also be considered as obstructing behavioral changes by maintaining their health behavior. However, health information providers can assist in promoting health behavior by identifying the subject's condition based on prior health related behaviors before acquiring desired information from consumers and providing customized information to facilitate behavioral change. Artificial intelligence can recommend relevant online content based on a user’s patterns of information search and health management experiences presents an opportunity to enhance support for health information seekers [7].

Perceived ease of use is a crucial independent variable that influences behavior intention. In the TAM proposed by Davis, he explains that in his research, perceived ease of use has an indirect influence on intention to use. Based on the model and the results of this study, it can be concluded that perceived ease of use given an HPM does not affect the intention to use but through perceived benefit indirectly [25].

The perceived usefulness of OHI influenced perceived benefit but did not impact perceived self-efficacy. Consumer behavior in utilizing the internet for health promotion is influenced by perceived ease of use, while those seeking disease-related health information are more affected by perceived usefulness [44,45]. In the original TAM by Davis, perceived usefulness, and perceived ease of use were significant factors affecting technology adoption [27]. In Korea, OHI seekers predominantly search for health information weekly, using around 1.78 mobile devices or personal computer applications. In this study, perceived usefulness demonstrated greater predictive power than perceived ease of use [25], although the latter significantly influence consumers, however, its impact in the context of HPB remains uncertain. The nature of influence depends on individual purposes, which supports the view that consumers evaluate health promotion-related content based on entertainment value and disease-related content based on practical value [46].

The variable of perceived benefit exhibited the most substantial explanatory power in the study, accounting for 27.5% of the variance, directly and indirectly influencing HPBs regarding behavioral intentions. Perceived benefits can be broadly categorized into internal and external benefits. For instance, internal benefits reduced nervousness and fatigue, while external benefits involve monetary rewards or social recognition [17]. Given the unprecedented nature of the COVID-19 pandemic, individuals experience heightened uncertainty about their health, often stemming from a lack of accurate health related knowledge [6]. Online resources minimize internal uncertainty by disseminating accurate information and enhancing the effectiveness of health related decision-making [1]. Moreover, health-promotion practices like physical activities, nutrition, and weight management shape consumers' intentions regarding concrete actual health behaviors [17].

The perceived ease of use of OHI also influenced perceived self-efficacy in behavioral skills. This aligns with the findings of previous studies indicating that the perceived ease of use of online patient portals positively impacts perceived self-efficacy in adults [47].

In this study, perceived self-efficacy can be interpreted as the belief that consumers can transfer the knowledge acquired through exploring OHI into behaviors. The results of this study suggest that perceived self-efficacy did not directly impact health promotion behaviors but exerted an indirect and significant influence through the behavioral intention to engage in health promotion behaviors. Among young adults, perceived self-efficacy was a significant predictor for seeking mental health counseling services [48].

The behavioral intention for health promotion behaviors directly impacted health promotion behaviors through the influence of motivation and behavioral skills factors. This direct and significant influence of intention on behavior was reaffirmed in this study, consistent with previous research in HPM and information motivation behavioral skills model, as well as in extended theories of planned behavior and planned behavior, where emotional responses to behavior are linked through intention to actual behavior [49]. Therefore, when providing health promotion-related content, it would be beneficial to thoroughly assess consumers’ intentions, such as asking a few questions with checklists. Setting differentiated goals based on these intentions can facilitate a smoother transition from intention to health promotion behaviors. This approach ensures that health promotion behaviors are naturally and effectively connected once intentions are enhanced. This study's findings align with research on determinants influencing health promotion behaviors in individuals at high risk of consumers or patients, where behavioral intention had a positive effect on HPB [50].

This study addresses a gap in the literature by elucidating the factors influencing behavioral intentions and HPB, focusing on motivational and behavioral skill factors to enhance consumers' self-engagement in health management. The findings underline the guidance that health information providers can derive from prior health related behavior, perceived ease of use, usefulness, perceived benefit, self-efficacy, and behavioral intention. Additionally, categorizing health information into general and disease-specific health information warrants consideration of various influencing factors, such as perceived benefit and perceived usefulness.

However, despite the robustness of the findings, this study has limitations. First, the subjects were adults willing to participate in both survey phases, possessing experience in seeking OHI. Generalizing these findings requires broader participation and further studies. Second, OHI use and HPBs relied on self-reporting through surveys, introducing potential recall bias and subjectivity. The study excluded variations in OHI use based on different characteristics of OHI services each participant used, focusing instead on subjective indices. Finally, the study found that the average age of the participants was 33.9 years old and nearly 98% of the sample has received a university-level education or higher. Therefore, premature generalization of the study's findings should be avoided. Further research is necessary to comprehensively understand how these effects vary across different consumer subgroups. Lastly, there have been significant advancements in digital technology since data collection. Although some time has passed, continuous impact of digital technology on HPBs reinforces the relevance of the data. Also, this research continues to hold significant value by providing baseline data for future comparisons and demonstrating methodological rigor, such as adherence to the Declaration of Helsinki.

## CONCLUSION

This study makes a novel contribution to the literature by identifying factors influencing behavioral intention and HPB of consumers of OHI based on the HPM and TAM. Among these factors, perceived benefits demonstrated the greatest explanatory power and directly affected the intention of health promotion. Notably, perceived usefulness was a key factor for consumers seeking disease-specific health information, whereas perceived ease of use was identified as a strong determinant for consumers seeking health promoting information. Consequently, to enhance consumers’ HPB, strategies should focus not only on behavioral intention but also on perceived benefit, perceived usefulness, prior health related behavior, perceived ease of use, and self-efficacy.

This study demonstrates a new perspective for eHealth consumers by offering important factors. Ultimately, the findings of this study will enhance the development of scientific theory and its practical application for eHealth consumers in the post-COVID-19 era.

## Figures and Tables

**Table 1.** Participant Characteristics and Health Information Technology in Health Promotion (N = 360)

Variables	Categories	n (%) or M ± SD
Age (yr)	20-29	162 (45.0)
	30-39	106 (29.4)
	40-49	37 (10.3)
	50-59	41 (11.4)
	≥ 60	14 (3.9)
		33.90 ± 12.30
Sex	Men	100 (27.8)
	Women	260 (72.2)
Occupation	Student	113 (31.4)
	Professional	138 (38.3)
	Office clerk	59 (16.4)
	Service or sales	14 (3.9)
	Other	36 (10.0)
Level of education	Less than high school	7 (1.9)
	College graduate	289 (80.3)
	Graduate school or higher	64 (17.8)
Household income per month in KRW10,000	≤ 249	43 (11.9)
	250-349	79 (21.9)
	350-449	74 (20.6)
	450-549	50 (13.9)
	≥ 550	114 (31.7)
		530.70 ± 493.50
Cohabitation	Family	244 (67.7)
	None (alone)	64 (17.8)
	Other	29 (8.1)
	Partner (relatives, friends)	23 (6.4)
Number of chronic diseases	None	242 (67.2)
	1-2	110 (30.6)
	≥ 3	8 (2.2)
Main method of using online resources	PC-based	161 (44.7)
	Mobile-based	199 (55.3)
Frequency of use	3 or more times/week	29 (8.1)
	1-2/week	100 (27.8)
	2-3/month	80 (22.2)
	1/month	71 (19.7)
	1/3-4 month	80 (22.2)
Number of websites or apps used	1	134 (37.2)
	2-3	181 (50.3)
	4-5	35 (9.7)
	≥ 6	10 (2.8)
		1.78 ± 0.75
Categories of health information	Health responsibility	143 (39.7)
	Physical activity	118 (32.8)
	Nutrition	52 (14.4)
	Spiritual growth	26 (7.2)
	Stress management	15 (4.2)
	Interpersonal relations	6 (1.7)
Purpose of using services	Seeking health information	250 (69.4)
	Online health counseling	36 (10.0)
	Appointment to see doctor	28 (7.8)
	Self-check	21 (5.8)
	Online consultation	12 (3.4)
	Join the health community	7 (1.9)
	Buying medical glossary	6 (1.7)
Inconveniences when using health information	Low credibility	181 (50.4)
	Too much information	89 (24.8)
	Insufficient consultation	38 (10.6)
	Insufficient information about diversity	21 (5.8)
	Difficulty searching	18 (5.0)
	Difficulty understanding	11 (3.1)
	Privacy and security	2 (0.3)

M = Mean; SD = Standard deviation.

**Table 2.** Descriptive Statistics of the Measured Variables

Variables	Number of items	Score range	M ± SD	Measured range	Skewness	Kurtosis	Reliability
Prior health-related behavior	6	6-30	18.07 ± 3.72	6-27	-0.22	-0.07	.63
Perceived ease of use of OHI	4	4-16	11.68 ± 2.02	5-15	-0.30	-0.20	.88
Perceived usefulness of OHI	4	4-20	13.78 ± 2.81	4-20	-0.09	0.19	.79
Perceived benefit	5	5-25	15.96 ± 5.96	5-25	0.20	0.05	.90
Perceived self-efficacy	3	3-15	10.91 ± 1.77	6-15	-0.18	0.23	.71
Behavioral intention	6	6-30	21.61 ± 3.47	12-30	-0.10	-0.05	.78
Health promoting behavior	20	20-100	65.07 ± 11.18	40-8	-0.13	0.76	.89

M = Mean; SD = Standard deviation; OHI = Online health information.

**Table 3.** Model Standardized Estimates and Predictive Effects in OHI Consumers (N = 360)

Endogenous variables	Exogenous variables	Standardized estimates	C.R.(*p*)	SMC	Standardized direct effect	Standardized indirect effect	Standardized total effect
Perceived benefit	Perceived OHI ease of use	.15	8.64 (< .001)	.28	.39	-	.39
	Perceived OHI usefulness	.12	4.59 (< .001)		.20	-	.20
	Prior health-related behavior	.07	1.39 (.163)		.07	-	.07
Perceived self-efficacy	Perceived OHI ease of use	.23	4.19 (< .001)	.15	.23	-	.23
	Perceived OHI usefulness	.07	1.24 (.215)		.07	-	.07
	Prior health-related behavior	.24	4.76 (< .001)		.24	-	.24
Behavioral intention	Perceived benefit	.76	6.49 (< .001)	.10	.76	-	.76
	Perceived self-efficacy	.12	1.89 (.059)		.12	-	.12
	Prior health-related behavior	.26	4.91 (< .001)		.26	.08	.34
Health-promoting behavior	Behavioral intention	.20	3.77 (< .001)	.15	.16	-	.16
	Prior health-related behavior	.25	4.55 (< .001)		.25	.08	.31
	Perceived benefit	.02	0.38 (.707)		.02	.12	.14
	Perceived self-efficacy	.09	1.59 (.113)		.09	.02	.11

OHI = Online health information; C.R. = Critical ratio; SMC = Squared multiple correlation.

## Data Availability

The data supporting the findings of this study can be obtained from the corresponding author upon reasonable request.
